# Severe Hyponatremia as the Initial Sign Preceding Guillain-Barré Syndrome, an Acute Inflammatory Demyelinating Polyneuropathy: A Case Report

**DOI:** 10.1155/2013/923602

**Published:** 2013-02-05

**Authors:** Benjamin Kloesel, LaTonya J. Hickson

**Affiliations:** ^1^Department of Medicine, Mayo Clinic, 200 First Street SW, Rochester, MN 55905, USA; ^2^Division of Nephrology and Hypertension, Department of Medicine, Mayo Clinic, 200 First Street SW, Rochester, MN 55905, USA

## Abstract

Guillain-Barré syndrome is an immune-mediated polyneuropathy that frequently presents with progressive muscle weakness. Hyponatremia has recently been described as a feature of this condition, generally appearing over the course of the illness and following the diagnosis of this demyelinating process. We report a case of Guillain-Barré syndrome presenting with severe hyponatremia that is further exacerbated by intravenous immune globulin therapy. Awareness should be raised for consideration of both Guillain-Barré syndrome and its treatment with intravenous immune globulin therapy as the cause of hyponatremia.

## 1. Introduction

Guillain-Barré syndrome (GBS) is an immune-mediated polyneuropathy characterized by progressive, symmetric muscle weakness (which is classically ascending) and depressed or absent deep tendon reflexes. GBS is a heterogeneous syndrome with at least four subtypes; the most frequently encountered form in North America and Europe is acute inflammatory demyelinating polyneuropathy (AIDP) [1]. Diagnosis can usually be established clinically and is supported by nerve conduction studies showing signs of peripheral demyelination and cerebrospinal fluid (CSF) exam with elevated total protein and normal CSF white blood cells (albuminocytologic dissociation). Renal function is usually not affected, but recent case reports have established a link between GBS and hyponatremia [2–5].

Hyponatremia of various forms is often encountered amongst hospitalized patients [6]. In euvolemic hyponatremia an increase in total body water is accompanied by normal total sodium and minimal to moderately increased extracellular fluid volume in the absence of edema. Euvolemic hyponatremia is commonly due to the syndrome of inappropriate secretion of antidiuretic hormone (SIADH). The normal regulation of antidiuretic hormone (ADH) secretion is mediated by the hypothalamus and autonomic nerve fibers. Damage to those structures from the autoimmune process in GBS can alter ADH release patterns leading to autonomic dysfunction and SIADH.

## 2. Case Presentation

A 62-year-old female with past medical history of hypertension, hyperlipidemia, and chronic obstructive pulmonary disease was admitted to the medical intensive care unit after being found unresponsive at home. Prior to this, she complained of malaise, decreased appetite, and mild dyspepsia that prompted an evaluation by her primary care provider on the day prior to admission. Significant findings on initial evaluation included obtundation with a Glasgow Coma Scale of 9, confusion, white blood cell count 18.1 × 10^9^/L, serum sodium 110 mmol/L (reduced from 136 mmol/L 3 months prior to admission), blood urea nitrogen 7 mg/dL, creatinine 0.4 mg/dL, serum glucose 118 mg/dL, serum osmolality 235 mOsm/kg, urine osmolality 413 mOsm/kg, random urine sodium 76 mmol/L, total cholesterol 161 mg/dL, and triglycerides 106 mg/dL. Serum protein electrophoresis was negative for monoclonal protein. Thyroid-stimulating hormone and 8 AM cortisol levels were unrevealing (1.0 mIU/L and 26 mcg/dL, resp.). Computed tomography (CT) scan of head and neck, chest X-ray, and electrocardiogram were normal. MRI showed multiple diffuse T2 signal changes in a nonspecific pattern without signs of osmotic demyelination. Electroencephalogram showed mild diffuse nonspecific slowing without epileptogenic foci. A lumbar puncture revealed one nucleated cell and mildly elevated total protein levels at 52 mg/dL. Bacterial cerebrospinal fluid (CSF) cultures and PCR for Cytomegalovirus, Epstein Barr Virus, and Herpes Simplex Virus remained negative. In the medical intensive care unit, empiric antibiotic coverage with ceftriaxone and levofloxacin was started and home medications including losartan and hydrochlorothiazide were held. Due to the severity of symptoms, infusion of hypertonic saline was initiated. Over the course of 24 hours the serum sodium level improved from a presenting value of 110 to 124 mmol/L.

**Figure 1:**
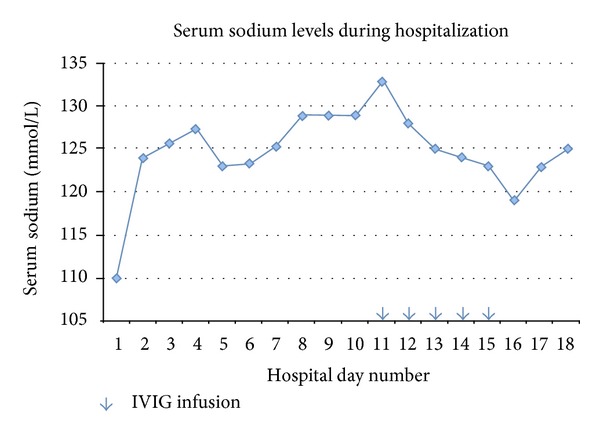
Serum sodium levels during the hospitalization. Arrows indicate IVIG doses given on the respective hospital days. Serum sodium levels were generally obtained in the morning. IVIG infusion was given in the evening.

With hypertonic saline infusion, the patient's sodium levels continued to slowly increase. On hospital day (HD) #4 the patient developed fever and leukocytosis. A chest X-ray showed a new area of consolidation suspicious for aspiration pneumonia for which broad spectrum antibiotics were started. The patient clinically improved and was transferred out of the intensive care unit on HD #5. Hypertonic saline had been discontinued. Management of SIADH was continued with fluid restriction and oral sodium chloride tablets. On HD #6 she complained of lower back pain; this was followed by numbness and tingling in both of her legs as well as gait instabilities. Neurologic examination revealed decreased deep tendon reflexes, muscle strength, and sensation to light touch in both lower extremities in addition to severe gait instability. An electromyogram was consistent with acute inflammatory demyelinating polyradiculoneuropathy. Treatment with a five-day course of a nonsucrose containing intravenous immune globulin (Privigen) therapy was initiated on HD #11. During this period, serum sodium levels temporarily decreased (Figure 1).

The patient was eventually discharged to a skilled nursing facility on HD #19 for outpatient physical therapy and rehabilitation. Hyponatremia management included fluid restriction and a higher sodium diet. Metoprolol and amlodipine were substituted for losartan and hydrochlorothiazide for blood pressure control. Over a five-month period, the residual bilateral lower extremity weakness slowly improved with physical therapy and laboratory studies revealed a normal serum sodium level (138 mmol/L). 

## 3. Discussion

The occurrence of hyponatremia in patients diagnosed with GBS is welldescribed [2–5]—in one prospective study of 50 patients diagnosed with GBS, hyponatremia was noted in 48% of cases [7] and motor dysfunction preceded the onset of hyponatremia. There have only been two prior case reports in which hyponatremia was observed prior to manifestation of neuromuscular deficits [8, 9]. 

While the underlying etiology of this phenomenon is presumably related to SIADH, there has been considerable discussion whether “pseudohyponatremia” from intravenous immune globulin (IVIG) administration is also playing a major role. 

Pseudohyponatremia usually occurs in the presence of increased lipid or protein concentrations when sodium is measured with indirect ion-selective electrodes. Lipids and protein occupy a part of the plasma sample whose aqueous fraction, along with the solutes, is excluded in indirect analytical methods, thereby lowering the sodium that is delivered for analysis. The problem can be avoided by the use of direct ion-selective electrodes [10]. While IVIG infusions are prone to causing pseudohyponatremia through delivery of large amounts of proteins [11], more recent studies have shown that the significant alterations in serum sodium levels cannot completely be explained by this mechanism [12]. One study examined the effect of IVIG infusion on serum sodium levels measured by direct ion-selective electrodes and found that hyponatremia was also present [13]; this “true hyponatremia” was related to osmotic translocation of water from the intracellular to the extracellular (intravascular) space mediated by an increase in intravascular osmolality secondary to sucrose-based IVIG infusion. 

In our patient, the initial hyponatremia responded to therapy but later relapsed in the presence of IVIG therapy. The IVIG formulation was free of sucrose. However, Privigen, currently marketed as the first and only IVIG formulation stabilized with proline, contains a nonessential amino acid which may also lead to hyponatremia. In this case, hyponatremia may be further induced by the amino acid acting as an osmolyte, thereby causing an osmotic water translocation [14]. The pathomechanism of hyponatremia in GBS has not been clearly elucidated, although multiple theories have been discussed including impaired autonomic nervous function involving the afferent fibers from vascular stretch receptors [5], resetting of the osmostat [15], increased sensitivity of distal tubular and collecting duct ADH receptors, and ADH-independent mechanisms [16].

Our patient case is unique in that severe hyponatremia preceded neurologic symptoms and the diagnosis of GBS. Signs and symptoms of GBS appeared during the hospital course following correction of the electrolyte disturbances. In most cases reported in the literature, hyponatremia was noted after a diagnosis of GBS was established. The question whether the hyponatremia might have been triggered by therapy with losartan and hydrochlorothiazide needs to be considered; given that the patient had been on a stable regimen with unchanged serial sodium levels prior to hospital admission, we feel that this explanation as the primary source of hyponatremia is less likely. 

In conclusion, this presentation raises the possibility that early changes in the autonomic nervous system triggered by GBS might lead to alterations in water and sodium balance that can precede symptomatic changes in the peripheral nervous system. Additionally, patients should be monitored for further hyponatremia induced by IVIG preparations when undergoing GBS therapy. Although a rare condition, both GBS and its treatment, IVIG, should be considered in the differential diagnosis of hyponatremia.
